# Optimization of* Ex Vivo* Murine Bone Marrow Derived Immature Dendritic Cells: A Comparative Analysis of Flask Culture Method and Mouse CD11c Positive Selection Kit Method

**DOI:** 10.1155/2018/3495086

**Published:** 2018-02-22

**Authors:** Rahul Ashok Gosavi, Sukeshani Salwe, Sandeepan Mukherjee, Ritwik Dahake, Sweta Kothari, Vainav Patel, Abhay Chowdhary, Ranjana A. Deshmukh

**Affiliations:** ^1^Department of Zoonosis, Haffkine Institute for Training, Research and Testing, Parel, Mumbai, India; ^2^Department of Biochemistry and Virology, National Institute for Research in Reproductive Health (NIRRH), Parel, Mumbai, India; ^3^Department of Virology and Immunology, Haffkine Institute for Training, Research and Testing, Parel, Mumbai, India; ^4^Department of Microbiology, Grant Medical College and Sir JJ Group of Hospital, Byculla, Mumbai, India

## Abstract

12–14 days of culturing of bone marrow (BM) cells containing various growth factors is widely used method for generating dendritic cells (DCs) from suspended cell population. Here we compared flask culture method and commercially available CD11c Positive Selection kit method. Immature BMDCs' purity of adherent as well as suspended cell population was generated in the decreasing concentration of recombinant-murine granulocyte-macrophage colony-stimulating factor (rmGM-CSF) in nontreated tissue culture flasks. The expression of CD11c, MHCII, CD40, and CD86 was measured by flow cytometry. We found significant difference (*P* < 0.05) between the two methods in the adherent cells population but no significant difference was observed between the suspended cell populations with respect to CD11c+ count. However, CD11c+ was significantly higher in both adhered and suspended cell population by culture method but kit method gave more CD11c+ from suspended cells population only. On the other hand, using both methods, immature DC expressed moderate level of MHC class II molecules as well as low levels of CD40 and CD86. Our findings suggest that widely used culture method gives the best results in terms of yield, viability, and purity of BMDCs from both adherent and suspended cell population whereas kit method works well for suspended cell population.

## 1. Introduction

Dendritic cells (DCs), the key initiators and modulators of primary immune response, bridge the adaptive and innate immune system and are crucial to elicit antigen specific immune responses. DCs, macrophages, and B cells are professional Antigen Presenting Cells (APC) whereas basophils, mast cells, eosinophils, and innate lymphoid cells are atypical APCs [1]. DCs stimulate both naïve and memory T cells [2, 3]. They capture and present foreign material on their surface and promote their differentiation into cytotoxic T lymphocytes (CTLs)/CD8^+^ and helper T cells (Th)/CD4^+^ cells [4]. In resting state, immature DCs are unable to process, capture antigen, and express low level of MHC and costimulatory molecules [5, 6]. DCs mature on activation by danger signals and upon infection of the host, leading to differentiation, maturation, and stimulation of naïve T cells [7, 8].

The potential of pulsed and antigen loaded DCs to initiate adaptive immune response has attracted major interest in vaccine research against infectious diseases and cancer [9, 10]. Novel DC isolation strategies are generally performed by DCs generated* in vitro *from murine bone marrow precursors [11, 12] and human monocyte precursors [13]. Prolonged cell culture in presence of specific growth factors and cytokines help DCs differentiate into murine bone marrow derived DCs (BMDCs) and human monocyte-derived DCs from their respective precursors. Murine myeloid DC (mDC) are CD11c^high^CD11b^high^B220^−^ whereas murine plasmacytoid (pDC) is CD11c^low^CD11b^−^B220^+^. Since both mDC and pDC are CD11c positive [14–16], CD11c is commonly used as a murine DC marker along with costimulator molecules like MHC II, CD40, CD80, and CD86 [17].

Generation of BMDCs by prolonged culture is based on the differentiation of DCs with either granulocyte-macrophage colony-stimulating factor (GM-CSF) alone [6] or in combination with Interleukin-4 (IL-4) [6, 18, 19]. More recently BMDCs have also been generated by Flt3L [20, 21] or Interleukin-3 (IL-3) [22] producing a mixture of CD8*α*+ and CD8*α*− pDC subsets. DC phenotype is dependent on concentration of cytokines and growth factors. In addition, other factors such as strain, age and gender of mouse, bone marrow dissection, culture medium, serum, and experimental time setup for the DC generation are also crucial for the generation and purification of BMDCs. Thus BMDC culture establishment is still carried out in a nonstandard and potentially suboptimal manner for many* in vitro *DC studies.

Classical 10–12-day procedure for generating BMDCs from murine bone marrow precursors is widely used method [11, 12]. Commercial kits for generation of BMDCs also give good cell yield, viability, and purity in 5-6 days efficiently with reduced chances of contamination. In this study, we optimized and compared the yield and purity of murine derived CD11c+ BMDCs cultured by flask culture method and CD11c Positive Selection kit based method, in the presence of low dose of GM-CSF in nontreated tissue culture flasks. The former is a routine culturing method using GM-CSF alone while the positive selection kit method is designed to isolate CD11c+ cells from single cell suspension of bone marrow precursors by positive selection beads.

## 2. Materials and Methods

### 2.1. Mice

7–10-week old BALB/c mice were maintained in a pathogen-free environment in the institute's animal house facility at 65–75°F and 40–60% relative humidity with 10–12 hr light-dark cycle. They were given autoclaved pelleted and fresh potable drinking water. All the experiments were approved by the Institutional Animal Ethics Committee (IAEC) and all procedures were as per the Committee for the Purpose of Control and Supervision on Experiments on Animals (CPCSEA) guidelines.

### 2.2. Chemicals/Reagents and Antibodies

Cell culture complete medium was comprised of RPMI 1640 (Life Technologies, India) with 10% heat inactivated and filtered (0.22 *μ*M, Merck Millipore, India) Fetal Bovine Serum (FBS) (Life Technologies, India); Penicillin-Streptomycin (100 U/ml, Sigma, USA), L-glutamine (2 mM, Sigma USA), 2-mercaptoethanol (50 *μ*M, Sigma USA), Hanks' Balanced Salt Solution (HBSS) (Sigma, USA); and phosphate buffered saline (PBS) (Gibco-Invitrogen, India). Recombinant-murine (rm) GM-CSF (conc. 0.2 mg/ml) was purchased from M/s BioLegend (San Diego, USA). Antibodies PE (phycoerythrin) conjugated CD11c (clone N148), PE/Cy7 conjugated MHCII IA/IE (clone M5/114), Alexa Fluor 488 conjugated CD86 (clone PO3), and PE/Cy7 conjugated CD40 (clone HM40-3) were obtained from M/s BioLegend (San Diego, USA).

### 2.3. Bone Marrow Preparation and Induction of Immature DCs

Male BALB/c mice were euthanized and femurs and tibiae were harvested and placed in 70% ethanol for 2-3 minutes for disinfection after removing extra tissues by Kleenex tissue paper. After rinsing off the ethanol with Hanks Balanced Salt Solution (HBSS), bones were transferred to RPMI-1640 in a sterile Petri dish. Bone ends were cut with sterile, sharp scissors, and the contents of marrow flushed with 2 ml of HBSS using a 29G × 1/2 needle syringe. By vigorous pipetting, bone marrow cells were diluted with HBSS to make homogeneous suspension. After 2 washes in HBSS, about 1 × 10^8^ total leukocyte counts were obtained per femur and tibia. BMDCs were generated as previously described [11, 12]. Briefly, according to cell count obtained, the cell pellet was resuspended in 10 ml complete RPMI 1640 medium. At day 0, cells were seeded in complete medium with rmGM-CSF (100 U/ml) in T25 tissue culture nontreated flask and incubated at 37°C with 5% CO_2_. At day 3, additional 5 ml complete medium with rmGM-CSF (80 U/ml) was added to the flasks and BMDCs were processed for the separation using either culture method or kit method.

### 2.4. Separation of BMDCs by Culture Method Using Low Doses of rmGM-CSF

On days 5, 7, 9, and 11, half of the culture supernatant was collected and centrifuged at 250*g* for 8 minutes and the cell pellet was resuspended in the same flasks with fresh 10 ml complete medium reducing the dose of rmGM-CSF (40 U/ml; 20 U/ml; 10 U/ml and 5 U/ml, respect.) to optimize the culture conditions. T25 flasks were observed microscopically for morphological changes like size and shape and results were recorded in every flask on days 0, 3, 5, 7, 9, and 10. Suspended and adherent cells were collected on day 12 from the medium and analyzed by flow cytometry for the further downstream processing.

### 2.5. Separation of BMDCs by EasySep Magnet-CD11c Positive Selection Kit Method (EasySep Kit)

On day 5, suspended and loosely adherent cells were harvested and isolated using EasySep kit (EasySep® Magnet, StemCell Technologies, Vancouver, Canada) according to the manufacturer's instructions. Briefly, desired cells were targeted with an antibody complex recognizing CD11c+ cells and dextran-coated magnetic particles. Labeled cells were separated using a magnet. Desired cells remained in the tube while unwanted cells were poured off. Cells were then analyzed by flow cytometry for immature DC proportion. Briefly, BMDCs were stained with their specific anti-mouse antibodies for 30 minutes at 4°C in dark and washed in FACS buffer (0.2% FBS-PBS).

### 2.6. Cell Yield, Viability, and Purity of the Separated BMDCs

On day 5 and day 12, immature cells were harvested from EasySep kit and T25 flasks, respectively, and cell yield was determined using the Trypan blue dye exclusion test (Sigma, USA) by the method of Rosenberg et al. [23]. The yield percentage was estimated by the formula: % yield = DC/total cell count × 100.

### 2.7. Assessment of BMDC Phenotype by Flow Cytometry

Separated BMDCs were washed with stain buffer and surface stained for DC markers. Dot plot analysis was carried out for DC surface markers. Dot plot of forward scatter (FSC) versus side scatter (SSC), FL1 versus FL2, and FL2 versus FL3 were drawn for each sample. Gating was adjusted for the analysis of desired cell population and debris was excluded by adjusting the threshold with low FSC and SSC properties. In each acquired sample, 10,000 events were recorded for further analysis of the data. Unstained control was used for the data analysis. Expression of costimulatory and surface markers was evaluated by FACS BD Accuri flow cytometer (Beckman Coulter, Fullerton, CA). Data were analyzed using Flowjo, LLC, Oregon, USA, software.

### 2.8. Statistical Analysis

Data were statistically analyzed using GRAPHPAD PRISM™ 5 software (Graph Pad software, Inc., La Jolla, CA). The representative experiments group differences were assessed using the paired *t*-test with *P* ≤ 0.05 being statistically significant. All the data were expressed as mean ± standard deviation (SD).

## 3. Results

### 3.1. Yield and Viability of BMDCs

The total cell population obtained from the fresh bone marrow harvested from femur and tibia ranged from 5.0 to 7.5 × 10^7^ cells/ml according to bone marrow used while the viability for day 0 was 95–98%. The yield and viability of BMDCs by culture method and kit method are mentioned in Table 1. It was observed that amongst both adhered and suspended cells, the highest yield and viability were obtained using culture method. Statistically significant difference (*P* < 0.01) was observed in the viability of adhered cells between the two methods (see supplementary data—comparison between adhered and suspended cell population—Figure A).

### 3.2. Morphological Characteristics of BMDCs in Culture

**Figure 1:**
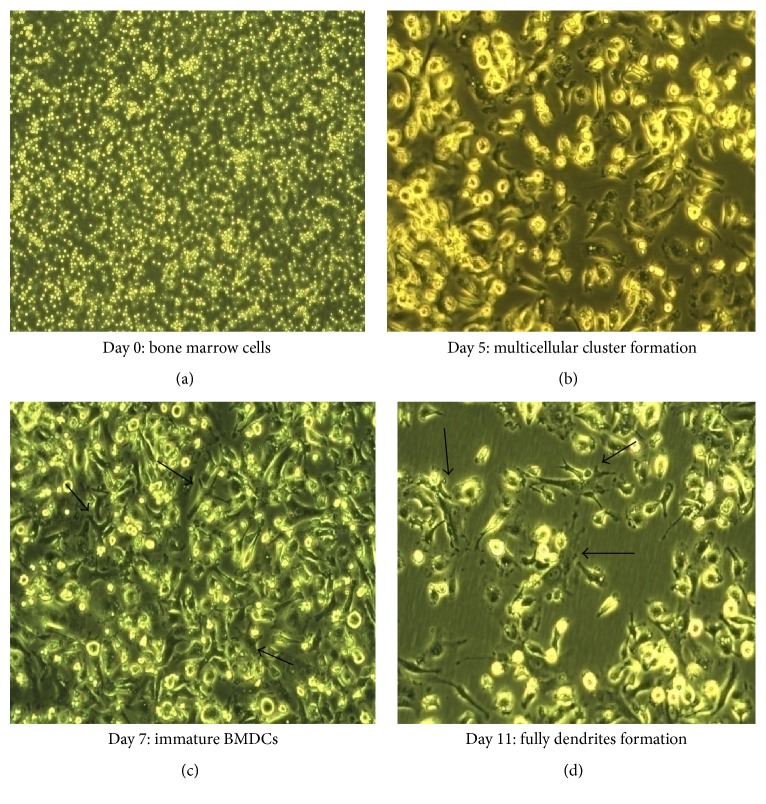
*Day wise morphological changes of immature BMDCs at low doses of rmGM-CSF.* Bone marrow cells were cultured in RPMI 1640 medium with 10% FBS containing low doses of rmGM-CSF and were observed under the light inverted microscope (magnification ×40). Black arrows indicate cells with a DC-like appearance. (a) Bone marrow precursors and total cells on day 0 with 100 U/ml rmGM-CSF; (b) day 5 BMDCs with 40 U/ml rmGM-CSF, showing colony and multicellulat clusters formation and early stages of dendrites; (c) day 7 with 20 U/ml rmGM-CSF immature BMDCs with ridgy shape, and (d) fully dendrites formation showing higher immature and lower mature BMDCs at day 11 with 5 U/ml rmGM-CSF.

BMDCs were cultured and induced with in complete RPMI 1640 medium supplemented with a given dose of rmGM-CSF. Microscopic examination of cultured BMDCs showed heterogeneous cell population. On day 0 with 100 U/ml rmGM-CSF, bone marrow progenitor cells showed spherical morphology (Figure 1(a)). Cells were small in size with defined cell membrane and in good health. On day 5, with 40 U/ml rmGM-CSF, spherical morphology with very early stages of “dendrites” formation on the cell surface was observed. Aggregated cells together with initiation of multicellular cluster formation could be seen at various sites (Figure 1(b)). Much higher percentage of nonadherent cells was observed by culture system.

Ridgy shaped BMDCs with a relatively smooth membrane surface were seen at day 7 with 20 U/ml rmGM-CSF, demonstrating that they are mainly in immature state (Figure 1(c)). Scanning electron microscopy gives clear idea about the shape and smooth surface membrane of immature BMDCs [24]. Converted adherent macrophages with elongated appendages and fibroblast-like appearance were also observed. Large sized colonies of BMDCs were formed at various sites showing large number of semiadherent and floating suspended cells. After prolonged culture, immature BMDCs become significantly larger in size and presented with a rough surface showing bigger and longer protrusions and pseudopodia (Figure 1(d)). Formation of this roughness, protrusion, and branched and extended morphology on the cell membrane in both adherent and suspended cells were considered to be mature and immature BMDCs.

### 3.3. CD11c+ DC Population

**Figure 2:**
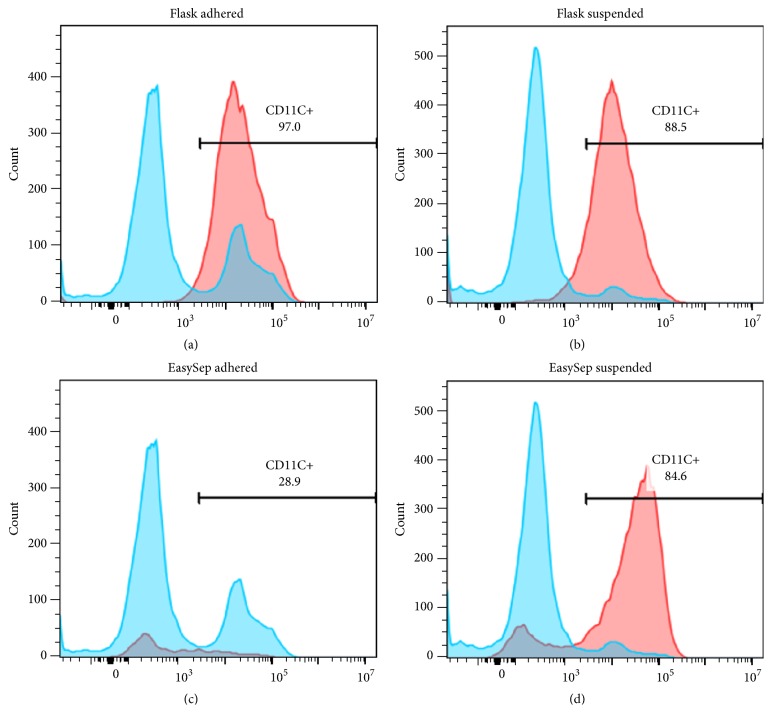
*Representative histogram plot *(*n* = 3)* for CD11c+ expression on BMDCs. *Bone marrow cells were cultured in RPMI 1640 medium with 10% FBS containing low doses of rmGM-CSF (as described in Materials and Methods) and harvested at day 12 for flask culture method and day 5 for EasySep Magnet Positive Selection kit based method. Untreated (sky blue) and low doses of rmGM-CSF treated (pink) BMDCs were stained with PE-labeled anti-CD11c and analyzed by flow cytometry. DCs were gated based on CD11c+ staining. Data were analyzed using Flowjo, LLC, Oregon, USA, software. Images show purity of CD11c+ cells with regard to percentages of adhered CD11c count generated by (a) flask culture method and (c) by EasySep Magnet Positive Selection kit based method and percentages of suspended cell population by (b) flask culture method and (d) by EasySep Magnet Positive Selection kit based method are indicated in the respective histograms (*n* = 3).

Culture and kit separated BMDCs were, respectively, surface stained for CD11c, a well-known DC specific marker, and analyzed by flow cytometry to determine the yield (Table 1). It was observed that highest (97%) purity was obtained in adhered cells by culture method; a marked difference in percentage of CD11c+ cell population was observed compared to EasySep kit method (Figure 2). CD11c+ cells were almost equal in both adherent and suspended cell population by culture method. However, by kit method at day 5, CD11c+ cells were enriched only in suspended cell population resulting in a statistically significant difference (*P* < 0.05) (see supplementary data—comparison between adhered and suspended CD11c+ cell population—Figure B).

### 3.4. Surface Marker Phenotype Analysis of Immature BMDC

**Figure 3:**
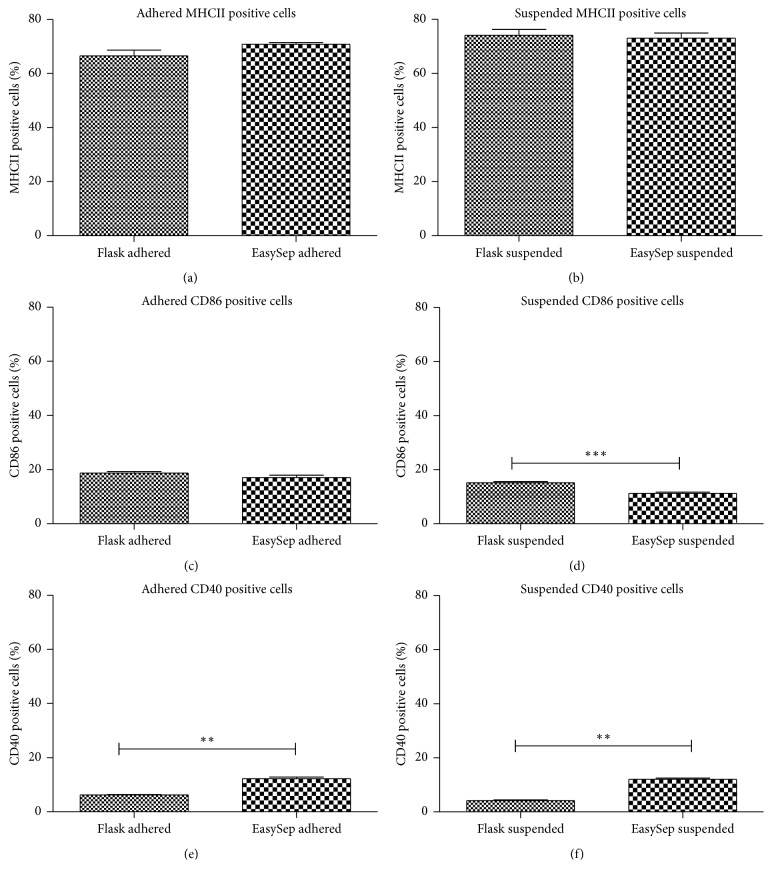
*Comparison between adhered and suspended BMDCs phenotype markers generated by flask culture method and EasySep Magnet Positive Selection kit based method.* The observed difference was statistically significant (*P* < 0.05) Graph showing the percentage of (a) MHCII positive cells expressing DCs in adhered cell population, (b) MHCII positive cells expressing DCs in suspended cell population, (c) CD86 positive cells expressing DCs in adhered cell population, (d) CD86 positive cells expressing DCs in suspended cell population, (e) CD40 positive cells expressing DCs in adhered cell population, and CD40 positive cells expressing DCs in suspended cell population. Results were analyzed with paired *t*-test and significant difference between the two methods of CD86 positive cells expressing DCs in suspended cell population and CD40 positive cells expressing DCs in both adhered and suspended cell population were seen (*P* < 0.05) but there was no significant difference between the MHCII positive cells expressing DCs in adhered and suspended cell population as well as in adhered CD86 positive cells expressing DCs in adhered cell population. ^*∗∗*^Statistically significant; ^*∗∗∗*^highly statistically significant.

**Figure 4:**
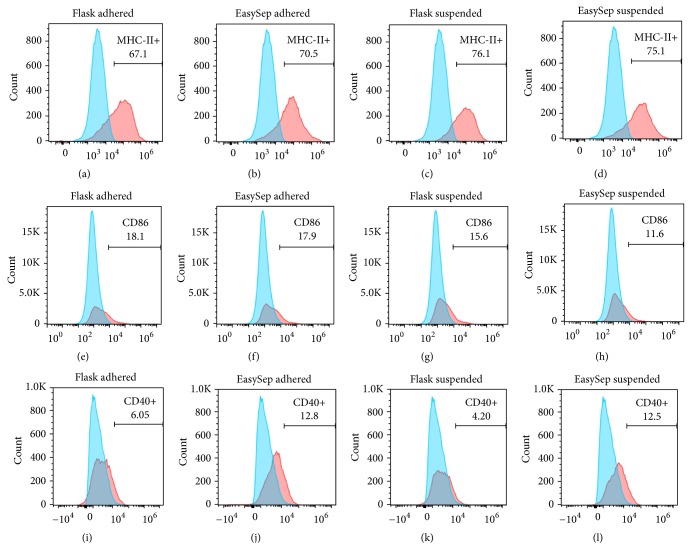
*Expression of surface markers of adhered and suspended rmGM-*CSF^low^* BMDCs generated in the absence of LPS.* FACS surface profile of surface markers BM-DCs from BALB/c mouse at day 12 for flask culture method and day 5 for EasySep Magnet Positive Selection kit based method. Bone marrow cells were cultured in RPMI 1640 medium with 10% FBS containing low doses of rmGM-CSF (as describe in Materials and Methods) and harvested, respectively, for above-mentioned methods. Untreated blank (sky blue) and low doses of rmGM-CSF treated (pink) adhered and suspended BMDCs were stained and analyzed by flow cytometry. Images show representative histograms (*n* = 2) of immature BM-DCs expressing cell population of adhered MHCII+ (a), suspended MHCII+ (c), adhered CD86+ (e), suspended CD86+ (g), adhered CD40+ (i), and suspended CD40+ (k) costimulatory molecules generated by flask culture method and adhered MHCII+ (b), suspended MHCII+ (d), adhered CD86+ (f), suspended CD86+ (h), adhered CD40+ (j), and suspended CD40+ (l) costimulatory molecules by EasySep Magnet Positive Selection kit based method.

Flow cytometry analysis showed that reduction of rmGM-CSF dose results in significant decrease in expression of costimulatory molecules. In our study, when rmGM-CSF was titrated down to 5 U/ml, the expression of surface markers also decreased. No statistical significance difference was observed between the two methods used to generate BMDC MHC II molecules (Table 1). The phenotype analysis of Immature BMDC revealed that GM-CSF^lo^ DC derived from culture as well as kit method expressed intermediate level of MHCII, decreased expression of CD86, and very low levels of CD40 (Figures 3 and 4).

## 4. Discussion

DCs can be used as immune adjuvant for antitumor therapies against malignancies and other infectious diseases. BMDCs are routinely employed in immune-modulatory therapies. Murine BMDCs are broadly used as* in vitro* model systems to investigate the role of DC for the evaluation of novel vaccines [25]. Several methods such as bone marrow culturing with either GM-CSF alone [11, 12] or in combination with IL-4 [6, 18], by Flt3L [20, 21] or IL-3 [22], are used for generation of murine BMDCs. Researchers have also worked on the influence of different culture mediums, cytokines, and bovine serum for generation of CD11c DCs [25–29].

In our study, the effects of two CD11c DC separation methods including flask culture method and EasySep kit method (commercially available Kit-Stem Cell Technologies) were compared regarding the production of immature BMDCs. Flask culture method is prolonged and produces cell population with high viability and purity in both adhered and suspended culture, compared to EasySep kit, which is rapid and produces abundant cell population in suspended cells only. Culturing of bone marrow cells on treated or nontreated tissue culture plates is also an important criterion for generation of DCs. Previous published protocols used tissue culture quality 24-well plates [11], bacterial quality 100 mm Petri dish [12], and nontreated polystyrene culture dishes [25] for generation of murine BMDCs with higher yield and purity of CD11c cells. In this protocol, we have used nontreated T25 flasks for prolonged culture method as well as for EasySep kit based method.

The generation of immature DC by the use of rmGM-CSF alone and with combination with IL-4 has been reported previously [6, 11, 12, 25, 26, 29]. However in this study the dose of rmGM-CSF used is lower than that reported by previously [6, 11, 12]. In order to optimize the dose of rmGM-CSF, we generated DCs by culturing bone marrow suspension at multiple doses at 100 U/ml, 80 U/ml, 40 U/ml, 20 U/ml, 10 U/ml, and 5 U/ml rmGM-CSF on 0, 3, 5, 7, 9, and 11 days, respectively, by flask culture method. The dose of rmGM-CSF for the EasySep magnet method was kept 100 U/ml and 80 U/ml for day 0 and day 3, respectively, and then harvested for downstream processing on day 5.

Differences were also observed in the CD11c+ DC population. Under culture conditions highest purity was obtained in both adherent and suspended cell population wherein CD11c+ cells were enriched only in suspended cell population by kit method. After 12 days of culturing, CD11c+ DCs ranged from 90 to 98%. The highest BMDC purity could be reached when adherent cell population were harvested and considered as a DC for downstream processing. Generally, after 8–10 days culturing with GM-CSF, the suspended cells are used as DC and cells adherent to culture system are excluded in order to increase purity of BMDCs [11, 12]. But in our experiment, for flask culture method, the semiadherent as well as suspended cell fractions contained a substantial portion of CD11c+ expressing BMDCs. These results were in agreement with Li and Lu [30], who demonstrated that adherent cells in GM-CSF culturing system of BMDCs are equally qualified DC as compared to suspended cells. Between the two methods used in our study, we found that both adhered and suspended cell population give higher CD11c+ BMDCs by prolonged culture method but only suspended cells give more CD11c+ BMDCs by EasySep kit method (Figure 2).

Granulocytes are major contaminants of suspended BMDCs and also respond to rmGM-CSF [12]. In our experiments, from day 3 onwards, they were visible as clusters of round cells (Figure 2(b)). Removing these clusters at the time of medium change bears the risk of removing the small clusters of expanding BMDCs. So we gradually reduced the dose of rmGM-CSF day 3 onwards and observed that majority of granulocytes did not persist longer than day 9-10 (Figure 2(d)). Our results are in agreement with the Lutz et al. who demonstrated that reducing the dose of rmGM-CSF in the culture method, majority of coevolving granulocytes disappear [6, 12]. However, in contrast, our protocol was able to achieve this effect without utilizing IL-4. Thus the proportion of BMDCs increased to more than 90% by replacing half of the complete medium with respect to rmGM-CSF dose and penicillin/streptomycin antibiotics. After day 9, cell purity, cell number, and the total viable cell count increased by culture method as compared to EasySep kit method (Table 1). The results of the present study demonstrate that prolonged culture increases the yield and purity of BMDC CD11c+ cells in both adherent and suspended population.

Differences in the costimulatory and surface markers can also exist within the CD11c+ population. Therefore, we decided to study the impact of both methods on surface and costimulatory molecules, which was accomplished through antibody staining against surface markers of immature DCs: MHCII, CD40, and CD86. With decreasing doses of rmGM-CSF, typically three different populations of cells have been reported, distinguished by their surface and costimulatory molecule's expression [6]. The MHC II^neg^  CD40^neg^  CD86^neg^ population were composed of granulocyte and myeloid precursor cells; MHC II^lo^ CD40^lo^ CD86^lo^ population differentiate into nonadherent DC and adherent macrophages referred to as immature DC and MHC II^hi^ CD40^hi^ CD86^hi^ population referred to as mature DC [6, 11, 12]. In our study, from the adhered and suspended population, MHC II was expressed in intermediate percentage amount in both methods (Figures 3 and 4).

Previous reports showed immature and mature cell population were generated from the bone marrow supernatant on the basis of low or high surface expression of MHC class II molecules [12]. In our study, when the amount of rmGM-CSF was reduced, the proportion of phenotypically immature DC also decreased. In a study by Lutz et al. [6], similar conditions of low rmGM-CSF and rm IL-4 resulted in obtaining phenotypically immature DC and functionally immature APC. At higher doses of rm GM-CSF, the phenotypes of these cells were generated and reported to be as an immature DC: MHCII^lo^ DC205^−^ CD11c^+^ CD80^+/−^ CD86^+/−^ and a mature DC: MHCII^hi^ DC205^+^ CD11c^+^ CD40^++^ CD80^+^ CD86^++^. Furthermore, it is reported that immature DCs are active in antigen uptake and processing but show only a moderate surface expression of MHC class II molecules and no or low levels of costimulatory molecules [6, 12]. Thus in our study, the culture conditions of low rmGM-CSF and no rm IL-4 supplementation yielded a population of cells that were mixture of phenotypically mature and immature (MHC intermediate) BMDCs. These results are comparable to Lutz et al. where cells were obtained by advanced culture method. Further these BMDCs exhibited very low levels of CD40 and CD86 cells (Figures 3 and 4). This may be due to presence of large number of immature cells. These procedures can be applied to produce functional DCs in future studies.

## 5. Conclusion

In conclusion, the present study is a preliminary comparison, based on CD11c and MHCII markers for the generation of immature BMDCs* in vitro* employing two widely used methodologies. To the best of our knowledge this is the first ever report of a comparative analysis of generation of BMDCs from adherent and suspended cell population in nontreated tissue culture flask using the conventional cell culture method and commercially available kit. Our findings suggest that conventional culture method gives the best results in terms of yield, viability, and purity of CD11c+ dendritic cell marker from both adherent and suspended cell population. It is an easy and cost-effective method for immature DC generation with a shortcoming of time required (12–14 days). The kit method works well for suspended cell population but it may not be recommended for BMDC generation from adhered cells. The advantage of kit based method is the enrichment of highly purified CD11c+ DC in a short time of 5-6 days; however the major limitation remains the cost of the kit and magnet itself. Functional assays are important for assessing the functionality of the cells generated. However, functional validation assay would be a necessary follow-up we aim to carry out in future studies. Also, the use of more surface and costimulatory markers would further aid the developing of a standard and efficacious method for* in vitro* generated mature BMDCs.

## Figures and Tables

**Table 1:** Phenotype comparison of adhered and suspended cell immature DCs population derived from flask culture method and EasySep Magnet Positive Selection kit based method.

Groups	Mean ± SD percentage of expression					
	Adhered cells			Suspended cells		
	Method I^a^	Method II^b^	*P* value	Method I^a^	Method II^b^	*P* value
Yield (cells/ml)	7.0–7.5 × 10^6^	6–7.0 × 10^6^	-	6.0–6.5 × 10^6^	6.0–6.5 × 10^6^	-
Total viable cells (%)	93.67 ± 1.528	73.33 ± 1.528	0.0019 (S)	83.33 ± 1.528	85 ± 1.000	0.1091 (NS)
CD11c+ (%)	96.70 ± 0.888	28.73 ± 0.862	0.0002 (S)	88.53 ± 1.150	88.80 ± 1.947	0.2757 (NS)
MHC II (%)	66.57 ± 2.150	70.87 ± 0.635	0.0502 (NS)	74.00 ± 2.207	72.97 ± 1.943	0.1106 (NS)
CD86 (%)	18.70 ± 0.655	17.13 ± 0.862	0.1508 (NS)	15.23 ± 0.404	11.23 ± 0.472	0.0002 (S)
CD40 (%)	6.21 ± 0.175	12.27 ± 0.550	0.0048 (S)	4.167 ± 0.251	12.10 ± 0.400	0.0016 (S)

^a^Method I: immature DCs derived from flask culture method. ^b^Method II: immature DCs derived from EasySep Magnet Positive Selection kit based method. Data were represented as mean ± SD and compared together using independent *t*-test; S: significant (*P* < 0.05); NS: nonsignificant.

## Supplementary Materials

Supplementary 1 Figure A: comparison between adhered and suspended cell population generated by flask culture method and EasySep Magnet Positive Selection kit based method as regards viability.

Supplementary 2 Figure B: comparison between adhered and suspended CD11c+ cell population generated by flask culture method and EasySep Magnet Positive Selection kit based method.
