# Supplementary material for: Optimization of Ex Vivo Murine Bone Marrow Derived Immature Dendritic Cells: A Comparative Analysis of Flask Culture Method and Mouse CD11c Positive Selection Kit Method
Source: Bone Marrow Res. 2018 Feb 22;2018:3495086. doi: 10.1155/2018/3495086 (PMC5842714; doi:10.1155/2018/3495086)
Supplement: Supplementary 1 — Figure A: comparison between adhered and suspended cell population generated by flask culture method and EasySep Magnet Positive Selection kit based method as regards viability. [file 3495086.f1.docx]

**Supplementary Figure A. Comparison between adhered and suspended cell population generated by Flask culture method and EasySep Magnet Positive selection kit based method as regards viability.** Graphic representation (n=3) of cell viability by Trypan blue dye exclusion test; (A) showing adhered cell viability and (B) showing suspended cell viability by both the methods. Results were analyzed by paired t-test and significant difference between the two methods in the adherent cells population was seen but there was no significant difference between the suspended cell populations with respect to cell viability (p<0.05).
