# Supplementary material for: Optimization of Ex Vivo Murine Bone Marrow Derived Immature Dendritic Cells: A Comparative Analysis of Flask Culture Method and Mouse CD11c Positive Selection Kit Method
Source: Bone Marrow Res. 2018 Feb 22;2018:3495086. doi: 10.1155/2018/3495086 (PMC5842714; doi:10.1155/2018/3495086)
Supplement: Supplementary 2 — Figure B: comparison between adhered and suspended CD11c+ cell population generated by flask culture method and EasySep Magnet Positive Selection kit based method. [file 3495086.f2.docx]

**Supplementary Figure B. Comparison between adhered and suspended CD11c+cell population generated by Flask culture method and EasySep Magnet Positive selection kit based method.** Results were analyzed with a paired t-test and significant difference between the two methods in the adherent cells population was seen but there was no significant difference between the suspended cell populations with respect to CD11c+ count. The observed difference was statistically significant (p<0.05). Graph showing (A) the percentage of CD11c+ adhered cell population and (B) the percentage of CD11c+ suspended cell population.
